# Effects of Transcranial Direct Current Stimulation (tDCS) in the Normalization of Brain Activation in Patients with Neuropsychiatric Disorders: A Systematic Review of Neurophysiological and Neuroimaging Studies

**DOI:** 10.1155/2020/8854412

**Published:** 2020-12-23

**Authors:** Melody M. Y. Chan, Yvonne M. Y. Han

**Affiliations:** Department of Rehabilitation Sciences, The Hong Kong Polytechnic University, Hong Kong, China

## Abstract

**Background:**

People with neuropsychiatric disorders have been found to have abnormal brain activity, which is associated with the persistent functional impairment found in these patients. Recently, transcranial direct current stimulation (tDCS) has been shown to normalize this pathological brain activity, although the results are inconsistent.

**Objective:**

We explored whether tDCS alters and normalizes brain activity among patients with neuropsychiatric disorders. Moreover, we examined whether these changes in brain activity are clinically relevant, as evidenced by brain-behavior correlations.

**Methods:**

A systematic review was conducted according to PRISMA guidelines. Randomized controlled trials that studied the effects of tDCS on brain activity by comparing experimental and sham control groups using either electrophysiological or neuroimaging methods were included.

**Results:**

With convergent evidence from 16 neurophysiological/neuroimaging studies, active tDCS was shown to be able to induce changes in brain activation patterns in people with neuropsychiatric disorders. Importantly, anodal tDCS appeared to normalize aberrant brain activation in patients with schizophrenia and substance abuse, and the effect was selectively correlated with reaction times, task-specific accuracy performance, and some symptom severity measures. *Limitations and Conclusions*. Due to the inherent heterogeneity in brain activity measurements for tDCS studies among people with neuropsychiatric disorders, no meta-analysis was conducted. We recommend that future studies investigate the effect of repeated cathodal tDCS on brain activity. We suggest to clinicians that the prescription of 1-2 mA anodal stimulation for patients with schizophrenia may be a promising treatment to alleviate positive symptoms. This systematic review is registered with registration number CRD42020183608.

## 1. Introduction

Neuropsychiatric disorders, such as schizophrenia, depression, and substance abuse disorders, are a collection of mental health conditions that are characterized by behavioral, emotional, and cognitive disturbances, which significantly affect the social and occupational functioning of an individual [1]. Together, these diseases are the top contributor to the global burden of nonfatal disease, reportedly accounting for approximately 20% in 2016 [2], and this number is expected to increase further in the future [3]. Despite the marked differences in etiology, abnormal brain activity is a common manifestation shared among these disorders [4, 5].

Among the many indicators used in different methods of measurement, event-related potentials (ERP) [6, 7] and blood-oxygen-level-dependent (BOLD) signals [8, 9] are two of the most commonly adopted indicators of brain activity. Compared to healthy individuals, people with neuropsychiatric disorders exhibit distinctive patterns of brain activity when these two groups are presented with the same stimuli/tasks that are believed to elicit task-relevant neural activation patterns. Regarding ERP, for example, people with schizophrenia have demonstrated consistently smaller P300 amplitudes than healthy individuals in various sustained attention tasks [10, 11] and the same has been shown in individuals with substance abuse disorders [12]; people with depression showed a reversed pattern of P100 amplitude changes when processing happy and sad faces and an enhanced N170 in facial recognition [13]. In fMRI studies, people with neuropsychiatric disorders commonly exhibited abnormal activation in the prefrontal cortex during basic cognitive and executive functioning tasks, such as a reduction in dorsolateral prefrontal cortex activation in schizophrenia patients during working memory tasks [14], a reduction in inferior frontal gyrus activation in people with attention-deficit/hyperactivity disorder (ADHD) in attentional control tasks [15], and an increase in right medial frontal cortex activation in people with depression during tasks requiring attention and memory manipulation [16]. Given that such abnormalities are well documented to be associated with impaired cognitive [17], social [18], and emotional [19] functioning, clinicians and researchers have attempted to normalize the brain activity patterns of these patients through different treatment methods.

Pharmacological treatments, such as antidepressants and antipsychotics, are currently the most common way of promoting normalization of brain activities. An fMRI meta-analysis of nine studies showed that antidepressants restored prefrontal cortex hypoactivation and reduced limbic system hyperactivation in patients with depressive disorders [20], whereas the activation of the anterior cingulate cortex and insular cortex was found to be modulated by antipsychotics in people with psychosis [21]. However, these medications are often associated with undesirable side effects, such as extrapyramidal side effects induced by not only first- but also second-generation antipsychotics [22], as well as hyponatremia, bleeding, or seizures induced by serotonin reuptake inhibitors (SSRIs) [23], which hinder treatment compliance [24, 25]. Alternatively, transcranial direct current stimulation (tDCS), hypothesized to be able to normalize brain activation abnormalities in patients with neuropsychiatric diseases, has been rigorously studied recently in terms of its proposed effects. tDCS is a noninvasive neuromodulation technique that utilizes the delivery of a weak direct current (usually under 3 mA) [26] through the scalp to the brain with the use of oppositely charged electrodes (i.e., anode and cathode) to alter the brain areas underneath the electrodes [27]. Early studies in healthy individuals showed the promise of tDCS in modulating neuroplasticity [28] and cortical excitability [29] in healthy individuals, and this treatment was later found to be able to promote motor recovery in stroke patients by modulating the abnormal neural activation patterns resulting from stroke [30]. Recently, the effects of tDCS on the modulation of cognitive function have been increasingly studied in healthy individuals and have yielded positive results [30], and it has been shown that changes in brain activity after tDCS are associated with improved cognitive performance [31]. These findings further reinforce the potential of tDCS to become a promising treatment modality for people with neuropsychiatric disorders, who often exhibit cognition-related deficits.

Indeed, some studies have revealed that tDCS could normalize brain activation in patients with neurological/neuropsychiatric disorders [32, 33]. However, the results are inconsistent with negative results reported previously [34, 35]. Moreover, in order for tDCS to be developed as a clinically relevant treatment regimen, neural changes must be associated with clinical gains, yet studies that reported such a brain-behavior relationship also revealed divergent results (see [36] for positive results but [37] for negative results for tDCS treatment in people with the same neuropsychiatric diagnosis). In order to clarify the brain-behavior relationships, a systematic review of randomized controlled trials, comparing the neural effects of tDCS across studies, could help fill this knowledge gap; no such review, however, is currently available. To fill this gap, we aimed to determine (1) whether tDCS could induce changes in brain activation in patients with neuropsychiatric disorders, (2) whether it normalizes or worsens participants' outcomes, and (3) whether the neurophysiological effects are correlated with clinical/behavioral outcomes.

## 2. Methods

### 2.1. Literature Search

This systematic review was performed according to the PRISMA guidelines [38] and was registered in the International Prospective Register of Systematic Reviews (PROSPERO; register ID CRD42020183608). A systematic literature search was carried out in March 2020 with the search terms “transcranial direct current stimulation”, “tDCS”, “functional magnetic resonance imaging”, “fMRI”, “electroencephalography”, and “EEG” in the electronic databases PubMed, Scopus, and Embase using title, abstract, and keyword searches (see Supplementary Materials for the actual search strategies for each of the databases). An additional search was performed one month before the submission (i.e., 20 June 2020) to ensure that all retrievable records were included. No limit was set on the publication dates. We also manually searched the bibliographies of related studies to identify possible articles to be included in this review.

### 2.2. Study Inclusion

Randomized controlled trials with tDCS administration on patients with neuropsychiatric disorders as defined in the Diagnostic and Statistical Manual of Mental Disorders (DSM-5) [1] with the ERP/brain blood flow outcome measured by EEG/fMRI were included in this review. We conducted three stages of screening to identify suitable records for inclusion in the systematic review. Duplicate records were first removed, after which we screened the titles and abstracts of the remaining articles to exclude studies without peer-reviewed empirical data (e.g., reviews, conference proceedings, book chapters, and editorials), nonhuman studies, studies that did not apply tDCS on patients with any type of neuropsychiatric disorder, studies that did not apply tDCS as the sole brain stimulation technique, studies where no EEG/fMRI measures were adopted, and studies without English full text. The third step was full-text screening of the remaining studies, which was conducted to exclude nonrandomized studies, studies without a sham tDCS control group, studies not measuring and presenting results regarding ERP and blood flow changes before and after tDCS, and studies that did not give between-group (i.e., active versus sham) comparisons that reflected tDCS effects. Two personnel (i.e., two research assistants: K.C. and A.C.) conducted the above screening separately. The second author resolved any discrepancies between the decisions made and provided the final judgement regarding the inclusion of studies.

### 2.3. Data Extraction

Two research assistants (P.H. and E.L.) extracted the demographic details (i.e., the numbers of participants in the sham and active tDCS groups as well as the participants' ages, psychiatric diagnoses, and medication status), tDCS protocol details (i.e., mode of stimulation, electrode size and montage, duration of stimulation, stimulation intensity, therapy/task accompanied by tDCS delivery, and relevant details), and outcome measures (i.e., experimental paradigm for recording ERP/cerebral blood flow, primary behavioral/clinical outcome results, and correlation between brain activity changes and clinical outcome). Information discrepancies in data extraction were confirmed and resolved by the first author. Electronic mails were sent to corresponding authors to ask for additional information/clarification if the data to be extracted were not complete.

### 2.4. Data Synthesis and Analysis

To determine whether tDCS outcomes from individual studies were appropriate to be pooled with meta-analytic techniques, we subjectively evaluate the clinical heterogeneity of patients, interventions, and outcomes, as well as the methodological heterogeneity in study design in all included studies; as recommended by Rao et al. [39], meta-analysis would not be conducted if either or both forms of heterogeneity were judged to be substantial. To address the question of whether tDCS induces changes in brain activation patterns in people with neuropsychiatric disorders, we provide an overall narrative synthesis of results. In order to address whether tDCS could normalize brain activation for different neuropsychiatric disorders, we first conducted a brief review of a previous meta-analysis regarding the abnormalities of brain activation in patients compared to healthy controls, such that we could determine whether the brain activity change induced by tDCS could be said to be a “normalization.” In order to explore whether the normalization effects brought by tDCS underlie behavioral/clinical improvements, narrative synthesis was conducted to summarize the brain-behavior relationship data reported in each of the included studies. If meta-analysis was deemed appropriate, effect size calculation and generation of the forest plot would be performed using Comprehensive Meta-Analysis (CMA; Biostat, Englewood, NJ) software; when test statistics could not be obtained from the corresponding authors but the results were described in text, nonsignificant and significant results would be assumed to have *p* values of 0.5 (1-tailed) and 0.05 [40], respectively. The risk of bias in individual studies was assessed by using the Cochrane Collaboration's tool [41] which was conducted by the first author and a research assistant (M. Cheng).

## 3. Results

### 3.1. Study Selection

**Figure 1:**
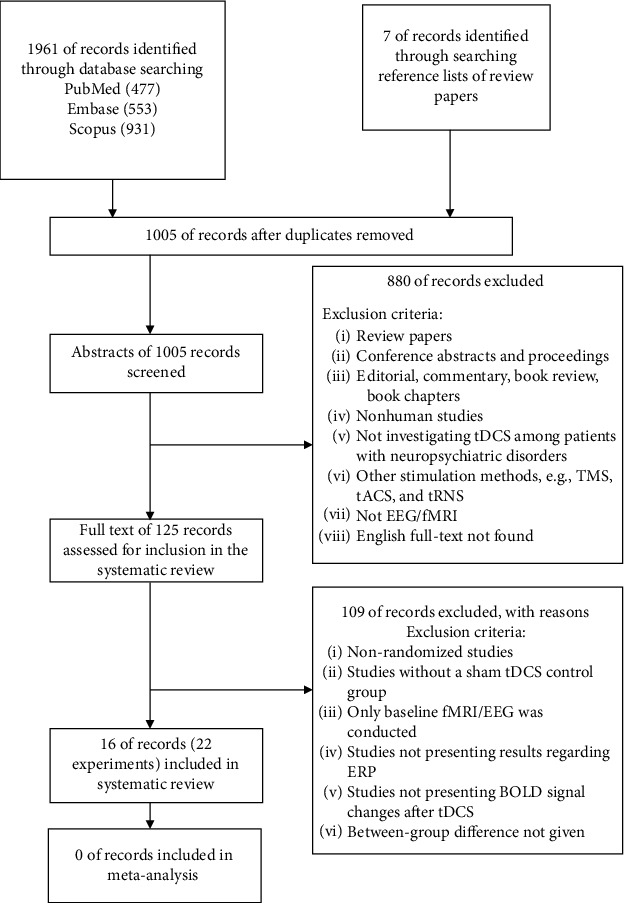
A flowchart illustrating the article screening process.

A total of 16 studies (with 22 experiments) were included in this review. The electronic database search yielded a total of 1968 studies, with 1005 records remaining for abstract screening after the removal of 963 duplicated records. 880 studies were excluded after exclusion criteria were applied at this stage. The full text of 132 records was further assessed for inclusion in the systematic review. A total of 109 studies were further excluded with additional exclusion criteria applied. See Figure 1 for the diagram illustrating the article screening procedure.

### 3.2. Study Characteristics

All of the experiments adopted prefrontal montage, except for experiments with temporal montage (experiments 1 and 2 from Rahimi et al. [42], experiment 1 from Impey et al. [43]) and one experiment investigating the effects of parietal montage (experiment 1 from Kim et al. [35]). The treatment duration for each session was 20 minutes for all studies except 15 minutes in den Uyl et al. [44] and 30 minutes in Orlov et al. [45]. Nine studies measured ERP, while the remaining seven studies investigated changes measured by fMRI. Seven studies investigated the effects of tDCS on brain activation in individuals with schizophrenia, and all of these studies involved patients with illness onset more than ten years with an average of 18.6 years [35, 43, 45–49]. Three studies investigated the effects of tDCS in people with substance abuse disorders [44, 50, 51]. One study investigated the effects of tDCS in individuals with depression [52]. A total of three studies investigated the effects of tDCS on neurodevelopmental disorders, with two on ADHD [53, 54] and one on dyslexia [42]. Two studies investigated MCI [33, 55]. The demographic details, tDCS protocols, clinical/behavioral outcomes, and brain-behavior relationship results are listed in Table 1. In view of the substantial clinical and methodological heterogeneity observed across the included papers, no meta-analysis was performed.

### 3.3. Risk of Bias

**Figure 2:**
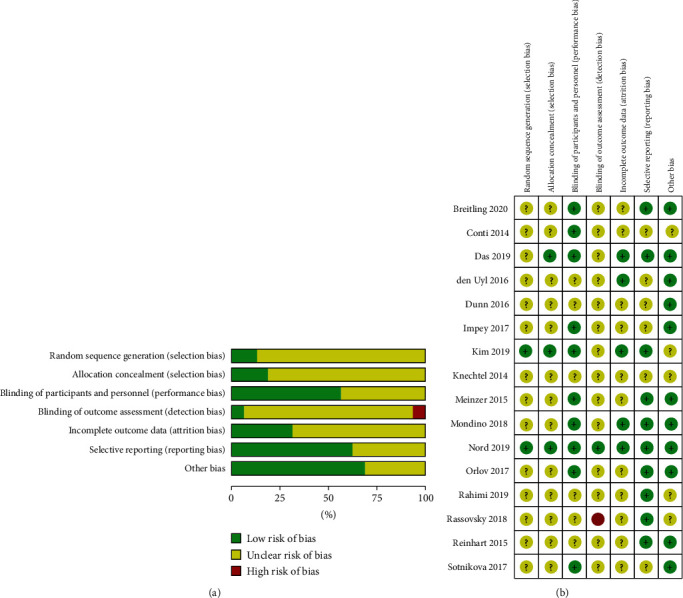
(a) A chart presenting authors' judgement as percentage about each risk of bias item across all included studies. (b) A chart showing authors' judgements about each risk of bias item for each included study.

With reference to Figure 2, more than half of the studies adopted adequate blinding procedures during treatment administration and reported all data from planned analysis to prevent reporting bias; for crossover studies, most of the studies adopted a washout period of more than two days to prevent carryover effects. However, most studies showed unclear bias in terms of random sequence generation, allocation concealment, blinding of outcome assessment, and incomplete outcome data.  Figure 2(a) displays the risk of bias items presented as percentages across studies, and Figure 2(b) shows the risk of bias summary for each included study.

### 3.4. Can tDCS Induce Changes in Brain Activation Patterns in People with Neuropsychiatric Disorders?

#### 3.4.1. ERP Studies

Five studies reported the effects of tDCS in modulating P300 amplitude [44, 46, 48, 51, 54]. All of these studies applied prefrontal stimulation (stimulating electrode placed over DLPFC, IFG, and supraorbital regions). Anodal stimulation was investigated in all of these studies, while the effects of cathodal stimulation were also studied in Dunn et al. [48] and Rassovsky et al. [46]. Overall, anodal tDCS was able to normalize P300 amplitude across these studies, while the effects of cathodal stimulation remained inconclusive. Three studies reported MMN amplitude changes [43, 46, 48]. Two studies adopted the prefrontal (DLPFC and supraorbital regions) montage, and the remaining study adopted the temporal montage [43]. Anodal stimulation was shown to reduce MMN amplitude, while cathodal stimulation remains inconclusive. Three experiments reported changes in N100 amplitude for anodal [42, 49]and bilateral [42] stimulation, showing that N100 was normalized by both stimulation modes, while Rahimi et al. [42] also reported a significant increase in P100 and P200 amplitude after either anodal or bilateral tDCS over the temporal region. Finally, anodal stimulation was also found to reduce the amplitude of N200 [54] but not for N170 [46], but cathodal stimulation could enhance the amplitude of N170 as stated in Rassovsky et al. [46].

#### 3.4.2. fMRI Studies

Seven studies investigated BOLD signal changes at the whole-brain level/*a priori* ROI after anodal tDCS over the prefrontal cortex when compared to sham-stimulated controls [33, 45, 50, 52, 53]. These experiments collectively suggested that anodal stimulation could increase BOLD signals not only over the brain regions directly under the stimulating electrode but also in regions remote from the expected stimulated areas. The remaining two studies reported between-group differences in changes in interhemispheric imbalance [35] and regional cerebral blood flow (rCBF) after active and sham tDCS, respectively. Kim et al. [35] reported that bilateral stimulation significantly normalized the interhemispheric imbalance in the active anodal stimulation group when compared to sham-stimulated individuals, while Das et al. [55] revealed an increase in rCBF in the right medial prefrontal cortex at rest after applying anodal stimulation over the left IFG.

### 3.5. Can tDCS Normalize Brain Activation in Different Patients with Different Neuropsychiatric Diagnoses?

A review of previous meta-analyses showing the aberrant brain activation patterns in patients with neuropsychiatric disorders included in this study is presented in Table 2.

#### 3.5.1. Schizophrenia

Among the 11 experiments, while seven experiments investigated anodal tDCS effects, two studies investigated cathodal and the remaining two investigated bilateral tDCS effects. With reference to previous meta-analyses and empirical studies, patients with schizophrenia were found to have reduced P300 [11, 57], N170 [58], N100 [59], MMN [60, 61], and ERN [62, 63] amplitudes when compared to healthy controls. Active anodal as well as bilateral tDCS stimulations were found to enhance the amplitudes of these ERP components [43, 46–49]. fMRI meta-analysis reviewed that while the medial frontal cortex was shown to have reduced activation during working memory tasks, the anterior cingulate cortex (ACC) was found to be hyperactivated during attentional control tasks in people with schizophrenia [14]; this phenomenon was also shown to be reversed by anodal tDCS [45]. For cathodal tDCS, the normalization effects remained inconclusive [46, 48].

#### 3.5.2. Substance Abuse

All three studies applied anodal stimulation. A previous meta-analysis showed that patients with substance abuse were found to have reduced P300 amplitude during auditory oddball tasks [12] and bilateral PCC activation reduction [64] at rest, which was found to be significantly enhanced after the application of anodal tDCS when compared to the sham control tDCS group [44, 50, 51].

#### 3.5.3. Depression

It was found that the left DLPFC was hypoactive in patients with depression, as reflected in a previous meta-analysis [65]. The sole study [52] investigating anodal tDCS in modulating brain activation for working memory and emotional face processing demonstrated that while there were no significant differences in DLPFC activation changes before and after the treatment between sham and active tDCS for working memory tasks, increased left DLPFC activation during the emotional face processing task reflected the normalization of brain activation for these patients.

#### 3.5.4. Neurodevelopmental Disorders

Previous meta-analyses revealed that ADHD patients showed reduced P300 amplitude [66], reduced DLPFC, SMA, PMC [67], and insula [68] activation, and enhanced precuneus [68] activation compared to their healthy counterparts. Anodal tDCS was shown to normalize aberrant brain activity, except for the enhancement of precuneus activation, which has already been shown to be enhanced in ADHD [53]. P300 was found to be enhanced regardless of the use of conventional or HD-tDCS, with the magnitude of enhancement being larger in HD-tDCS, although the difference in magnitude does not reach statistical significance [54]. Regarding dyslexia, other empirical studies except Rahimi et al. [42] have identified P100 [69] and N100 [70, 71] amplitude abnormalities, although the direction of effects remained inconclusive, as no meta-analysis could be identified. After anodal tDCS, it was found that P100, N100, and P200 amplitudes were reduced, although it remains debatable whether these changes reflect normalization.

#### 3.5.5. Neurodegenerative Disorders

Although meta-analyses were not available, two reviews reported a decrease in resting cerebral blood flow (CBF) and reduced activation in the inferior frontal gyrus in patients with MCI compared to healthy individuals. Anodal tDCS was found to enhance prefrontal CBF [55], reflecting a normalization effect, but it was also found to reduce activation in the bilateral IFG [33], which ran counter to normalization.

### 3.6. Brain-Behavior Relationship

Eight of the 16 included studies reported results of the correlations between changes in brain activity and behavioral/clinical outcomes after tDCS. When reaction time (RT) performances in memory [45] and learning [43] tasks were investigated as a behavioral indicator, significant correlations between reduction in RT with increased activation in the left dorsolateral prefrontal cortex (DLPFC) and increased frontal mismatch negativity (MMN) amplitude were reported. For on-task accuracy performance, although eight experiments reported between-group differences, only three experiments reported brain-behavior correlations; in Orlov et al. [45], the same attentional control task (i.e., the Stroop task) was given during tDCS stimulation and pre-/post-tDCS assessments and increased activation in the anterior cingulate cortex (ACC) significantly correlated with accuracy improvement before and after tDCS. For other experiments in which the assessment and treatment tasks were nonidentical, nonsignificant correlations were found between accuracy results and changes in activation in the anterior cingulate cortex for an untrained semantic memory retrieval task [33], as well as in working memory task [52]. When the relationship between psychiatric symptom changes and brain activity after tDCS was studied, Reinhart et al. [47] reported significant correlations between an increase in error-related negativity (ERN) and a reduction in the severity of delusional symptoms, while the correlation between changes in the severity of depressive symptoms and DLPFC/ACC activation [52], as well as the relationship between changes in the frequency of addictive behaviors and the right posterior cingulate gyrus (PCC) [50], was nonsignificant. Three studies investigated the correlations between score changes in standardized neurocognitive [46, 55], sociocognitive [46], and metacognitive [35] assessments with brain activity, and nonsignificant relationships were reported for all of these experiments.

## 4. Discussion

This systematic review was aimed at investigating the effects of tDCS in normalizing aberrant brain activities among people with neuropsychiatric disorders. After conducting a comprehensive literature search by browsing electronic databases and manual searches from the reference lists of relevant studies, 16 studies with 22 experiments that studied tDCS effects with ERP or fMRI activation measures were included in this systematic review. With converging evidence from both neurophysiological and neuroimaging studies, tDCS was shown to be able to induce changes in brain activation patterns in people with neuropsychiatric disorders. Importantly, anodal tDCS appeared to normalize aberrant brain activation in patients with schizophrenia and substance abuse, with this effect being selectively correlated with reaction times, task-specific accuracy performance, and some symptom severity measures. We first discuss the normalization effects and treatment implications in schizophrenia and other neuropsychiatric disorders, followed by an account regarding the phenomenon observed for the brain-behavior relationship.

### 4.1. Brain Activity Normalization in Schizophrenia and Other Psychiatric Diagnoses: Treatment Implications and Possible Research Development

Across all psychiatric diagnoses, the brain activity normalization effects of tDCS were most studied in patients with schizophrenia. In particular, prefrontal tDCS showed the most evidence of normalizing brain activity across different ERP and fMRI parameters that were identified to be aberrant in these patients in previously published meta-analytic data. Notably, these results can be generalized only to patients with chronic schizophrenia, given that the included population had mean illness duration of 18.6 years. Although the brain normalization effect was statistically significant, the majority of accuracy and reaction time performance in cognitive tasks showed nonsignificant improvements after the treatment. This was consistent with the nonsignificant behavioral findings reported by a meta-analysis of single-session tDCS in healthy individuals [72]. There are three possible reasons to explain this. First, and probably the most common problem existing in the current tDCS literature on patients with neuropsychiatric disorders, is the lack of power of the included studies to detect behavioral changes. Second, previous behavioral research suggested that there are interindividual variabilities in response to tDCS; while some might benefit from tDCS, some participants might actually show impaired cognitive performance after tDCS [73–75]. Indeed, another study included in this review by Nord et al. [52] reported that there are neural predictors that determine the behavioral treatment outcome; for instance, they reported that pretreatment activation of the left DLPFC was positively associated with posttreatment depressive symptom improvement. Collectively, these results imply that tDCS might be suitable only for some of the patients to optimize treatment gain and the decision of who would benefit and who would not depend on our understanding of the neural predictors, which is currently in the very early stages of research. Third, the lack of pairing with a cognitive task (e.g., working memory training) during tDCS delivery might contribute to the nonsignificant behavioral gains despite the significant neural gains. A previous meta-analysis has shown that concurrent working memory training could promote a small but significant effect of DLPFC anodal stimulation [76]. However, what kind of task should be administered and how it should be administered are some of the key questions to be studied, especially for cognitive enhancement with tDCS, given that previous reports have shown that anodal tDCS *per se* could facilitate or inhibit cortical excitability, which depended solely on the speed of the motor task being performed [77].

Regarding the neural effects of tDCS on other psychiatric diagnoses (i.e., substance abuse disorders, ADHD, dyslexia, depression, and MCI), we observed that tDCS tends to demonstrate the normalization effects as well, but more studies have to be performed regarding each of the individual diagnosis to yield conclusive results; additionally, for some diagnoses in which the abnormal brain activity is still under debate (e.g., inconclusive results in N100 amplitude between patients with dyslexia and healthy controls [42, 70, 71]), tDCS might be regarded as a tool to probe neural activity [78] and enhance our understanding of these diseases in the future, rather than as a treatment.

### 4.2. Selective Correlation of Brain Normalization with Behavioral/Clinical Measures

It appears that, in the first place, the brain activity changes were not significantly correlated with behavioral/clinical outcomes in the majority proportion of studies, making it hard to see the clinical relevance of tDCS, which is considered one of the prerequisites for introducing tDCS to become a promising treatment regimen in daily clinical practice. However, it was observed from this review that only some indicators reflecting brain activity changes correlate with particular parameters, namely, the correlation of reaction times with MMN and left DLPFC activation during memory and learning tasks, accuracy rates of trained tasks administered during tDCS stimulation, and ERN amplitude changes with particular disease severity measures. We examined this phenomenon by understanding the possible neuronal mechanisms of tDCS. Increasing direct evidence from animal studies has shown that tDCS could moderate NMDAR-dependent synaptic plasticity (see Cavaleiro et al. [79] for a review), and human magnetic resonance spectroscopy (MRS) studies showed that tDCS could modulate the concentration of gamma-aminobutyric acid (GABA), a neurotransmitter acting at inhibitory synapses in the brain [80]. This translational evidence leads to the hypothesis that tDCS might bring about specific behavioral changes by moderating synaptic plasticity of the stimulated brain regions as well as the functionally connected networks [81]. Given the established relationships between (1) GABA and reaction time [82], (2) GABA and MMN [83], and (3) MMN and synaptic plasticity [84, 85], we could interpret the significant brain-behavior relationships between RT and brain activation changes in [43, 45] as indirect evidence showing the effects of tDCS in modulating synaptic plasticity. Following the above proposition, when tDCS stimulation was directly applied to the core brain regions underlying a specific psychiatric symptom, e.g., delusion, a psychiatric symptom that has been recently found to be underlain by the deficits of the cognitive control circuit with ACC being the core neural correlate [86], significant brain-behavior relationships could be expected using the appropriate biomarker (i.e., ERN has been recognized as an electrophysiological index of ACC activation [87–89]) to reflect brain activity changes and a sensitive assessment tool that reflects clinical changes, as documented by Reinhart et al. [47].

On the other hand, the relationships between tDCS-induced brain activity changes, accuracy, and other standardized cognitive measures appeared to be mediated by the presence of the highly specific task during stimulation. Many empirical studies have demonstrated the task-specific effects of tDCS aimed at enhancing memory [90], learning [91], and other higher-order cognitive functions [92, 93]. From computational modeling [94] and animal studies [95, 96], it has been shown that the electric field induced by tDCS is low (below 1 V/m) at a stimulation intensity between 1 and 2 mA, resulting in “subthreshold” (rather than “supratheshold” stimulation applied by transcranial magnetic stimulation) neuromodulatory effects over ongoing neural processes. In other words, tDCS preferentially modulates the task-activated network; without the concurrent tasks guiding the stimulation effects, tDCS might not recruit the targeted network, for example, the aberrant neural network associated with various types of neuropsychiatric illness. Indeed, when we compared the significant correlation between accuracy performance in the emotional attentional control (Stroop) task and ACC resulting from presenting the same experimental paradigm before, during, and after tDCS as reported by Orlov et al. [45], with other studies given nonidentical training during tDCS administration [33, 52], it might be possible that due to the recruitment of different networks during pre-/post-treatment EEG/fMRI assessments when compared to the brain network recruited during therapy sessions, brain-behavior correlations could not be established. This would bring about another issue: does it mean the transfer of tDCS cognitive enhancement effects might be very limited, given that only highly specific tasks induce brain changes that are correlated with behavioral changes? In fact, previous research has demonstrated the potential of repetitive, task-relevant tDCS administered on consecutive days to promote cognitive skill transfer, which can last for nine months among healthy subjects [90]. Although studies that applied repetitive tDCS in our current review did not show significant correlations with accuracy and scores from standardized cognitive assessments [52, 55] given the small sample size of each study and the limited number of repetitive tDCS studies available, future research might further investigate the longitudinal effects of repetitive tDCS in establishing brain-behavior relationships, an increasingly studied issue that potentially supports tDCS as a clinically relevant option for neurorehabilitation.

## 5. Limitations

Although we planned to conduct a meta-analysis for each separate neuropsychiatric diagnosis if the number of articles met the *a priori* threshold set by the power analysis, such analysis was not conducted due to the limited number of studies available; instead, only systematic review was conducted to address our enquiry. In addition, the exclusion of non-English articles and data published in other publication genres (e.g., conference abstracts, letters and commentaries, and thesis) might limit the generalizability of our review. Furthermore, it should be noted that the majority of papers included in this review did not explicitly report the procedures for random sequence generation, allocation concealment, and the blinding of assessors; hence, selection and detection biases might be induced and influenced the validity of results. Regarding the data availability, although we have contacted the corresponding authors for the studies that provide insufficient information for our analyses, we did not receive their reply before the data analysis, or even before this manuscript is submitted. Furthermore, we found that brain-behavior correlations were not reported in seven studies, and our analysis of this relationship could be based only on the available significant and nonsignificant results. Future studies might consider the investigation of brain-behavior correlations such that the clinical relevance of tDCS application could be further understood, which could in turn benefit the development of novel treatments for patients with neuropsychiatric disorders.

## 6. Conclusion

This systematic review was aimed at investigating the effects of tDCS in normalizing aberrant brain activities among people with neuropsychiatric disorders as well as the clinical relevance of tDCS regarding its effects in moderating brain activations. With convergent evidence from both neurophysiological and neuroimaging studies, tDCS was shown to be able to induce changes in brain activation patterns in people with neuropsychiatric disorders. Anodal tDCS appeared to normalize aberrant brain activation in patients with some psychiatric diagnoses, namely, schizophrenia and substance abuse disorders. The detection of brain-behavior correlations in some specific measures but not others might imply a need for careful consideration of the choice of behavioral measurements, as well as therapy/task design that engages the appropriate cognitive neuronal networks, to improve the clinical relevance of tDCS. Such improvements will be an important factor determining the fate of tDCS in neuropsychiatric practice in the future.

## Figures and Tables

**(a):** 

Study [first author (year)]	Patient characteristics				Experimental details						Outcome measures		
	Diagnosis	Total *N*	Age group	Concurrent mediation	Mode^†^	Montage^‡^	Duration (min)	Total number of sessions	Intensity (mA)	Therapy/task accompanied by tDCS	Modality (EEG/fMRI): paradigm	Behavioral/clinical findings (*α* = 0.05; active vs. sham)	Brain-behavior relationship (*α* = 0 .05)
Schizophrenia													
Kim (2019) [35]	Schizophrenia	11	Adult	Yes	Expt 1: bilateral	Anode: P4 Cathode: P3	20	1	2	Nil	fMRI: illness awareness task	fMRI illness awareness task (i) Level of insight: n.s.	Correlations between interhemispheric imbalance in CBF and clinical scores: n.s.
		11			Expt 2: bilateral	Anode: F4 Cathode: F3	30	1	2	Nil	fMRI: illness awareness task		
Orlov (2017) [45]	Schizophrenia	24	Adult	Yes	Anodal	Anode: F3 Cathode: Fp2	30	1	2	Working memory task (*n*-back)	fMRI: working memory task (*n*-back)	fMRI *n*-back task (i) RT: n.s.	Significant correlation between the 2-/3-back performance 1 day after tDCS and the increased activation in L DLPFC (*p* < 0.05)
										Stroop task	fMRI: stroop task	fMRI stroop task (i) ACC: n.s.	Significant correlation between accuracy performance in the incongruent condition activation in the ACC (*p* < 0.005)
Rassovsky (2018) [46]	Schizophrenia	37	Adult	Yes	Expt 1: anodal	Anode: F3 Cathode: Fp2	20	2	2	Nil	EEG: auditory oddball task	Clinical assessment performance (ACC) (i) Speed of processing: n.s. (ii) Working memory: n.s. (iii) Verbal memory: n.s. Reasoning and problem solving: n.s.	Correlation between EEG results from the active group and cognitive scores: n.s.
											EEG: emotion recognition task		
		37			Expt 2: cathodal	Cathode: F3 Anode: Fp2	20	2	2	Nil	EEG: auditory oddball task		
											EEG: Emotion recognition task		
Reinhart (2015) [47]	Schizophrenia	17	Adult	Yes	Anodal	Anode: FCz Cathode: R cheek	20	1	1.5	Nil	EEG: feedback-based learning task	EEG feedback-based learning task (i) RT: n.s.	Gain in ERN amplitude correlated with lower delusion score (*p* < 0.0001)
Dunn (2016) [48]	Schizophrenia	24	Adult	Yes	Expt 1: anodal	Anode: Fp1, Fp2 Cathode: R upper arm	20	2	1	Nil	EEG: auditory oddball task	Not stated	Not stated
											EEG: passive attention auditory duration deviant paradigm		
		24			Expt 2: cathodal	Cathode: Fp1, Fp2 Anode: R upper arm	20	2	1	Nil	EEG: auditory oddball task		
											EEG: passive attention auditory duration deviant paradigm		
Impey (2017) [43]	Schizophrenia	12	Adult	Yes	Expt 1: anodal	Anode: between C5 and T7 Cathodal: Fp2	20	1	2	Nil	EEG: auditory oddball task	Behavioral working memory task (2-back) (i) RT: n.s. (ii) ACC: n.s.	Not stated
		12			Expt 2: anodal	Anode: F3 Cathode: Fp2	20	1	2	Nil		Behavioral working memory task (2-back) (i) RT: *p* < 0.05 (active < sham) (ii) ACC: *p* < 0.05 (active > sham)	Greater frontal MMN change correlated with faster RT (*p* < 0.05)
Knechtel (2014) [49]	Schizophrenia	14	Adult	Yes	Anodal	Anode: F3 Cathode: Fp2	20	1	2	Nil	EEG: go/no-go task	EEG go/no-go task (i) ACC: n.s.	Not stated
Substance abuse													
Mondino (2018) [50]	Tobacco abuse	24	Adult	No	Anodal	Anode: between F4 and Fp2 Cathode: between O1 and T5	20	10	1	Nil	fMRI: visual oddball task (smoking-related vs. neutral)	Reduction in craving: *p* < 0.05 (active > sham)	R PCC increase in activation does not correlate with changes in cigarette consumption: n.s.
den Uyl (2016) [44]	Alcohol abuse	39	Adult	No	Anodal	Anode: F3 Cathode: Fp2	15	3	1	Nil	EEG: visual oddball task (alcohol-related image vs. neutral image)	Reduction in craving: n.s.	Not stated
Conti (2014) [51]	Crack-cocaine abuse	13	Adult	No	Bilateral	Anode: F4 Cathode: F3	20	1	1	Nil	EEG: visual oddball task (crack-related image vs. neutral image)	Not stated	Not stated
Depression													
Nord (2019) [52]	Depression	39	Adult	No	Anodal	Anode: F3 Cathode: L deltoid	20	8	1	60-minute cognitive behavioral therapy with senior CP (symptom relief)	fMRI: working memory task (*n*-back)	fMRI *n*-back task: (i) RT: n.s. (ii) ACC: n.s.	Correlation between increased bilateral DLPFC activation and *n*-back improvement: n.s.
											fMRI: emotional processing task	(i) fMRI emotional processing task (ii) ACC: n.s.	Correlation between amygdala activation and emotional processing task: n.s.
Neurodevelopmental disorders													
Sotnikova (2017) [53]	Attention deficit/hyperactivity disorder	16	Adolescent	No	Anodal	Anode: F3 Cathode: Cz	20	1	1	Working memory task (*n*-back)	fMRI: working memory task (*n*-back)	fMRI *n*-back task ACC: <0.05 (active > sham)	Not stated
Breitling (2020) [54]	Attention deficit/hyperactivity disorder	15	Adolescent	No	Expt 1: anodal	Anode: F8 Cathode: Fp1	20	1	1	Working memory task (*n*-back)	EEG: working memory (*n*-back)	EEG *n*-back task (i) ACC: n.s. (ii) RT: n.s.	Not stated
		15			Expt 1: anodal (HD-tDCS)	Anode: F8 with 4 surrounding cathodes	20	1	0.5	Working memory task (*n*-back)		EEG *n*-back task (i) ACC: n.s. (ii) RT: n.s.	
Rahimi (2019) [42]	Dyslexia	17	Children	No	Expt 1: anodal	Anode: T7 Cathode: R shoulder	20	1	1	Nil	EEG: gap detection task	(i) EEG gap detection task temporal perception: *p* < 0.001 (active > sham)	Not stated
		17			Expt 2: bilateral	Anode: T7 Cathode: T8	20	1	1	Nil		(i) EEG gap detection task ACC: *p* < 0.001 (active > sham)	
Neurodegenerative disorders													
Das (2019) [55]	Mild cognitive impairment	16	Elderly	No	Anodal	Anode: F7 Cathode: R shoulder	20	8	2	Reasoning and inferencing strategy training	fMRI: resting	Clinical assessment performance (ACC) (i) Task switching: *p* < 0.05 (active > sham) (ii) Strategic learning: *p* < 0.05 (active > sham) (iii) Episodic memory: *p* < 0.05 (active > sham)	Correlation between regional CBF and clinical improvement: n.s.
Meinzer (2015) [33]	Mild cognitive impairment	18	Elderly	No	Anodal	Anode: F7 Cathode: Fp2	20	1	1	Semantic memory task	fMRI semantic word retrieval task	fMRI semantic word retrieval task (i) ACC: *p* < 0.05 (active > sham)	Correlation between changes in activation and reduction in errors: n.s.

^†^Mode of stimulation was classified based on a previously published framework [56]. ^‡^Montage location was reported according to the EEG 10-20 system; the anatomical positions were reported for extracephalic montage. Expt: experiment; *N*: number of participants; EEG: electroencephalography; fMRI: functional magnetic resonance imaging; L: left; R: right; n.s.: nonsignificant (at *α* = 0.05 significance level); RT: reaction time; ACC: accuracy; ACC: anterior cingulate gyrus; PCC: posterior cingulate cortex; DLPFC: dorsolateral prefrontal cortex; ERN: event-related negativity; MMN: mismatch negativity; HD-tDCS: high-definition transcranial direct current stimulation; CBF: cerebral blood flow.

**Table 2:** Abnormal brain activation of patients with neuropsychiatric disorders when compared to healthy controls.

Diagnosis	EEG/fMRI indicator	Task	Increase/decrease when compared to controls	Meta-analytic reference (if applicable)
Schizophrenia	P300	Auditory oddball	Decrease	Bramon et al. (2004) Qiu et al. (2014)
	N170	Face processing	Decrease	McCleery et al. (2015)
	N100	Paired click paradigm	Decrease	Rosburg (2018)
	ERN^†^	Attentional control	Decrease	Foti et al. (2012) Mathalon & Ford (2012)
	MMN	Auditory discrimination	Decrease	Umbricht & Krlijes (2005) Erikson et al. (2016)
	MFC activation	Working memory	Decrease	Glahn et al. (2005)
	ACC activation	Attentional control	Increase	Glahn et al. (2005)
Substance abuse	P300	Auditory oddball	Decrease	Euser et al. (2012)
	PCC activation	Resting	Decrease	Xiao et al. (2015)
Depression	L DLPFC activation	Working memory, emotional face processing	Decrease	Groenewold et al. (2013)
Attention deficit/hyperactivity disorder	P300	Auditory oddball	Decrease	Szuromi et al. (2011)
	DLPFC activation	Working memory, attention	Decrease	Cortese et al. (2012)
	SMA activation		Decrease	
	PMC activation		Decrease	
	Insula activation		Decrease	Hart et al. (2012)
	Precuneus activation		Increase	
Dyslexia^†^	P100	Auditory	Increase	Rahimi et al. (2019) Araujo et al. (2015), right brain
	N100		Increase	Rahimi et al. (2019) Helenius et al. (2002)
			Decrease	Bonte & Blomert (2004)
	P200		Increase	Rahimi et al. (2019)
Mild cognitive impairment^‡^	Prefrontal resting CBF	Resting	Decrease	Hays et al. (2016)
	IFG activation	Semantic memory retrieval	Decrease	Nellessen et al. (2014)

^†^Meta-analysis/review not available; results from empirical studies were reported. ^‡^Meta-analysis not available; results from systematic review were reported. EEG: electroencephalography; fMRI: functional magnetic resonance imaging; ERN: event-related negativity; MMN: mismatch negativity; MFC: medial frontal cortex; ACC: anterior cingulate gyrus; PCC: posterior cingulate cortex; L: left; DLPFC: dorsolateral prefrontal cortex; SMA: supplementary motor area; PMC: premotor cortex; IFG: inferior frontal gyrus; CBF: cerebral blood flow.

## Supplementary Materials

Supplementary Materials Literature search strategies applied for different electronic databases in this review.
