# Supplementary material for: Effects of Transcranial Direct Current Stimulation (tDCS) in the Normalization of Brain Activation in Patients with Neuropsychiatric Disorders: A Systematic Review of Neurophysiological and Neuroimaging Studies
Source: Neural Plast. 2020 Dec 23;2020:8854412. doi: 10.1155/2020/8854412 (PMC7773462; doi:10.1155/2020/8854412)
Supplement: Supplementary Materials — Literature search strategies applied for different electronic databases in this review. [file 8854412.f1.docx]

**Supplementary information: Literature search strategies applied for different electronic databases in this review**

1. PubMed

| **Search** | **Keywords** |
| --- | --- |
| #1 | (“transcranial direct current stimulation*”[Title/Abstract] OR tDCS[Title/Abstract]) |
| #2 | (“functional magnetic resonance imaging”[Title/Abstract] OR fMRI[Title/Abstract]) |
| #3 | (electroencephalogra*[Title/Abstract] OR EEG[Title/Abstract]) |
| #4 | #1 AND #2 |
| #5 | #1 AND #3 |
| #6 | #4 OR #5 |

1. Embase

| **Search** | **Keywords** |
| --- | --- |
| #1 | ‘transcranial direct current stimulation*’:ti,ab,kw OR tdcs:ti,ab,kw |
| #2 | ‘functional magnetic resonance imaging’:ti,ab,kw OR fmri:ti,ab,kw |
| #3 | electroencephalogra*:ti,ab,kw OR eeg:ti,ab,kw |
| #4 | #1 AND #2 |
| #5 | #1 AND #3 |
| #6 | #4 OR #5 |
| #7 | #6 AND **‘article’**/it |

1. Scopus

TITLE-ABS-KEY (("transcranial direct current stimulation*" OR tdcs) AND ("functional magnetic resonance imaging" OR fmri OR electroencephalogra* OR eeg)) AND (LIMIT-TO (PUBSTAGE, "final")) AND (LIMIT-TO (DOCTYPE, "ar")) AND (LIMIT-TO (EXACTKEYWORD, "Human")) AND (LIMIT-TO (LANGUAGE, "English"))
